# The effect of different types of physical activity on cognitive reaction time in older adults in China

**DOI:** 10.3389/fpubh.2022.1051308

**Published:** 2022-11-24

**Authors:** Yujie Liu, Xiao Hou, Zhengyan Tang, Hanyue Zhang, Jingmin Liu

**Affiliations:** ^1^Department of Sports Science and Physical Education, Tsinghua University, Beijing, China; ^2^Lang Ping Research Center for Sports Culture and Policy, Beijing Normal University, Beijing, China; ^3^School of Sport Science, Beijing Sport University, Beijing, China; ^4^Key Laboratory of Sports and Physical Health Ministry of Education, Beijing Sport University, Beijing, China; ^5^School of Physical Education, North East Normal University, Changchun, China

**Keywords:** reaction time, cognitive function, physical activity, old adults, aging

## Abstract

**Introduction:**

Aging is not only reflected in the degeneration of physiological functions but is also embodied in the decline of psychological and cognitive functions. The decline of cognitive function can reduce the quality of life in older adults, and even potentially cause Alzheimer's disease, which may lead to a heavy burden on patients, families, and society. The purpose of this study was to investigate the effects of physical activity (PA) on cognitive reaction time in older adults.

**Methods:**

A cross-sectional survey design was used in this study. A total of 839 elderly subjects were recruited from Beijing and Shanghai. In total, 792 subjects met the inclusion criteria (age > 60 years, without disability, speech, and hearing impairment), including 384 men (age:67.7 ± 5.7 years) and 408 women (age: 68.2 ± 5.8 years). The PA was assessed by the Physical Activity Scale for the Elderly (PASE). All kinds of PA were divided into three levels: “Low” (< 50% average score), “Moderate” (50–150% average score), and “High” (>150% average score). The reaction time of subjects was measured by the selective reaction tester (Model: CSTF-XF, TFHT, Beijing, China).

**Results:**

For leisure-time PA, the results showed that the cognitive reaction time of older adults in the “Low” group (1.11 ± 0.32 s) was significantly longer than that in the “Moderate” group (1.05 ± 0.30 s, *p* < 0.01) and the “High” group (0.99 ± 0.28 s, *p* < 0.01). For housework PA, there was no significant difference in the cognitive reaction time among the three groups (“Low”: 1.09 ± 0.31 s; “Moderate”: 1.07 ± 0.31 s; “High”: 1.05 ± 0.28 s, *p* > 0.05). For work-related PA, the results showed that the cognitive reaction time of older adults in the “Low” group (1.09 ± 0.30 s) was significantly longer than that in the “High” group (0.99 ± 0.28 s) and the “Moderate” group (1.03 ± 0.32 s, *p* < 0.01).

**Conclusion:**

The PA has a positive effect on reducing cognitive reaction time in older adults. It is recommended that older adults maintain a moderate level of leisure PA and work-related PA to delay the decline in cognitive reaction time.

## Introduction

Population aging is a major issue facing human society in the twentifirst century, and it is also one of the more serious social problems in China. The WHO states that by 2025, there will be 1.2 billion people over the age of 60, 75% of whom will be in developing countries (1). The latest survey shows that the proportion of China's population aged 60 years and above has reached 18.70%, with the proportion of people aged 65 years and above reaching 13.50%, which is close to the standard share of a deeply aging society (14%) (2). Faced with the reality that China's elderly population is large, and the aging process is significantly accelerating, it is especially important to maintain the physical and mental health of the elderly.

Cognitive function in older adults is one of the key indicators of physical and mental health and is closely related to their quality of life. On the one hand, with aging, the elderly experience varying degrees of decline in organismal function and a consequent increase in health problems, leading to a decline in physiological and immune function (3). On the other hand, during the aging process, nerve cells in the elderly gradually atrophy or even apoptosis, leading to a decrease in neurophysiological reserve and causing a decline in cognitive function in the elderly. Neuroscience studies have shown that dopamine is an important neurotransmitter in the hippocampus that regulates the excitability of the cerebral cortex, but as we age, the concentration of dopamine decreases, impairing cognitive function in humans (4), and those who are severe enough develop diseases, such as Alzheimer's disease. In addition, cognitive decline is more strongly correlated with poor health outcomes, such as basic activities of daily living impairment (5), low quality of life (6), and death (7). Therefore, it is important to improve the level of cognitive function in older adults to maintain their health and quality of life.

Physical activity (PA) is a human activity that is highly directional, subjective, and consciously produced by skeletal muscle contraction. Since the 1970s, research on the benefits of PA for cognitive function in older adults has received increasing attention. PA has become the focus of research to slow cognitive decline, improve executive function, promote neural growth in the brain, and reduce the risk of dementia in older adults. Previous studies have found that older adults who regularly participate in a variety of physical activities have a 30–46% lower risk of cognitive decline and a 28–45% lower likelihood of developing Alzheimer's disease compared to those who are sedentary and less active (8). An organized, individualized, high-intensity, long-term, and multi-element exercise program maintains cognitive performance in older adults (9). Studies have also found negative effects of reduced PA levels on cognitive function. Rogers et al. (10) did a follow-up analysis of changes in PA levels and cognitive function in 90 older adults over 4 years and found that those subjects with reduced PA levels had reduced cerebral blood flow and reduced cognitive function.

Maintaining PA as an important means of preventing and controlling various chronic diseases provides new directions for improving cognitive function in older adults. PA can improve a variety of cognitive functions in older adults by increasing cardiorespiratory fitness (11), such as cognitive processing speed (12), memory (13, 14), executive function (12, 15), and basic perceptual function (16). However, the overall concept of PA is broad and includes a variety of activities of different natures, such as leisure PA, housework, and work-related PA (17). The effect of different categories of PA on cognitive improvement may not be the same. Most of the current studies are limited to the effects of total PA or leisure PA on cognitive function(10, 18–21). The effects of housework and work-related PA on cognitive function are rarely studied (22). Therefore, this study aims to investigate the effects of different types of PA on cognitive function in older adults to provide a reference for achieving active aging.

## Methods

### Subjects

A cross-sectional health survey design was used in this study. A total of 839 subjects were recruited from the community in the Beijing and Shanghai areas. Inclusion criteria are as follows: age > 60 years, clear consciousness, basic reading and writing ability, and no significant speech or hearing impairment. All tests were completed in 1 day, and subjects were asked to fill out a questionnaire about their illness and physical condition to ensure they met the inclusion criteria for this study. The final number of elderly subjects included in the study was 792, of whom 384 were men (age: 67.7 ± 5.7 years) and 408 were women (age: 68.2 ± 5.8 years).

All subjects were informed of the detailed procedures and signed the informed consent documents before participating in the study. Additionally, subjects were clearly informed that they could withdraw from the study at any time for any reason, and all information would be kept anonymous and confidential. The study was approved by the Medical Ethics Committee of Tsinghua University.

### PA assessment

Physical activity was measured by the internationally classic Physical Activity Scale for the Elderly (PASE). The PASE questionnaire was developed by Washburn at Illinois at Urbana-Champaign University in 1993 (17), which consists of leisure PA, housework, and work-related PA. Leisure PA includes walking, light sport and recreational activities (such as Tai Chi, yoga, golf, and fishing), moderate sport and recreational activities (such as doubles tennis, table tennis, and dancing), strenuous sport and recreational activities (such as jogging, swimming, and cycling), and muscle strength exercise, and each PA is assessed on a 4-point scale of “days of activity per week” (never, 1–2, 3–4, and 5–7 days) and “time of activity per day” (<1, 1–2, 2–4 h, and more than 4 h). Housework includes cleaning, gardening, repairing electrical appliances, and taking care of others. Work-related PA mainly includes paid work and volunteer work. The items of housework activity and work-related PA were presented by asking the subjects whether they had performed any activity in the past week, and the subjects answered “yes/no.”

The PASE involves 13 questions with a total score of 0–360. The final score was calculated by adding the weighted scores of the 12 items (the first item “sedentary activity” was excluded), with higher scores indicating greater PA. The PASE is widely used in many countries, and it has good reliability and acceptable levels of validity (reliability: 0.897 and validity: 0.442) among the elderly in China (23). The questionnaire-based survey process had strict quality control.

### Reaction time assessment

The reaction time can reflect the information processing speed and evaluate the cognitive function (24). The reaction times of subjects were measured by the selective reaction tester (Model: CSTF-XF, TFHT, Beijing, China). During the test, the subject pressed the red “start” button with the middle finger of the dominant hand and waited for the signal to be issued, and then pressed the signal key as fast as possible. After the signal disappeared, the subject pressed and held the “start” button again with the middle finger and waited for the next signal to be given, a total of 5 times. After the subject had answered the signal five times, all the signal keys would emit “light” and “sound” signals at the same time, indicating the end of the test. The instrument automatically recorded the average reaction time of five times, and the record was in seconds, retaining two decimal places. All subjects should be clear about the test process before the test and the best scores were chosen between the two tests.

### Statistical analysis

According to the average score of each part of the PASE, all kinds of PA were divided into three levels: “Low” (< 50% average score), “Moderate” (50–150% average score), and “High” (>150% average score). Results were expressed as mean ± SD for continuous variables or frequencies (percentages) for categorical variables. An independent-samples Test was used for comparison between the two groups. PASE scores did not conform to a normal distribution, and the Mann–Whitney *U*-test was used for comparison between groups. The one-way ANOVA and Tukey's *post hoc* tests were conducted to compare the difference in cognitive reaction time among older adults with different PA levels of a certain PA type and to identify the optimal PA level in the specific PA type for improving cognitive reaction time. The level of significance was set at *p* < 0.05. In this study, we also indicated the significance level at *p* < 0.01. The statistical analyses were implemented by using SPSS 26.0.

## Results

### Demographic analysis

The descriptive characteristics and PA levels of the 792 subjects are shown in Table 1. More than half of the older adults had insufficient levels of leisure PA, 70% had low levels of work-related PA, and most of the older adults had moderate to high levels of housework.

**Table 1:** Descriptive characteristics and physical activity (*n* = 792).

**Characteristic**	**Participants**	**Leisure PA Level**			**Housework PA Level**			**Occupational PA Level**		
		**Low**	**Moderate**	**High**	**Low**	**Moderate**	**High**	**Low**	**Moderate**	**High**
**Participants**	792	436 (55.1)	220 (27.8)	136 (17.2)	193 (24.4)	449 (56.7)	150 (18.9)	565 (71.3)	135 (17.0)	92 (11.6)
**Gender**
male	384	216 (56.3)	107 (27.9)	61 (15.9)	80 (20.8)	206 (53.6)	98 (25.5)	271 (70.6)	72 (18.8)	41 (10.7)
female	408	220 (53.9)	113 (27.7)	75 (18.4)	113 (27.7)	243 (59.6)	52 (12.7)	294 (72.1)	63 (15.4)	51 (12.5)
**Age (years)**
60~74	692	406 (58.7)	186 (26.9)	100 (14.5)	167 (24.1)	393 (56.8)	132 (19.1)	486 (70.2)	121 (17.5)	85 (12.3)
≥75	100	30 (30.0)	34 (34.0)	36 (36.0)	26 (26.0)	56 (56.0)	18 (18.0)	79 (79.0)	14 (14.0)	7 (7.0)
**Education level**
Primary school or below	57	40 (70.2)	11 (19.3)	6 (10.5)	17 (29.8)	32 (56.2)	8 (14.0)	44 (77.2)	7 (12.3)	6 (10.5)
Junior high school	365	220 (60.3)	102 (27.9)	43 (11.8)	86 (23.6)	220 (60.3)	59 (16.2)	258 (70.7)	64 (17.5)	43 (11.8)
Senior high school	208	120 (57.7)	57 (27.4)	31 (14.9)	56 (26.9)	110 (52.9)	42 (20.2)	148 (71.2)	37 (17.8)	23 (11.1)
Vocational colleges	76	30 (39.5)	21 (27.6)	25 (32.9)	16 (21.1)	39 (51.3)	21 (27.6)	50 (65.8)	17 (22.4)	9 (11.8)
University	86	26 (30.2)	29 (33.7)	31 (36.0)	18 (20.9)	48 (55.8)	20 (23.3)	65 (75.6)	10 (11.6)	11 (12.8)
**Occupation**
Mental worker	238	98 (41.2)	75 (31.5)	65 (27.3)	59 (24.8)	125 (52.5)	54 (22.7)	171 (71.8)	37 (15.5)	30 (12.6)
Light worker	430	266 (61.9)	112 (26.0)	52 (12.1)	105 (24.4)	259 (60.2)	66 (15.3)	307 (71.4)	78 (18.1)	45 (10.5)
Heavy worker	106	60 (56.6)	30 (28.3)	16 (15.1)	23 (21.7)	57 (53.8)	26 (24.5)	71 (67.0)	19 (17.9)	16 (15.1)
Others	18	12 (66.7)	3 (16.7)	3 (16.7)	6 (33.3)	8 (44.4)	4 (22.2)	16 (88.9)	1 (5.6)	1 (5.6)
**Smoking**
Never	568	305 (53.7)	160 (28.2)	103 (18.1)	153 (26.9)	329 (57.9)	86 (15.1)	407 (71.7)	97 (17.1)	64 (11.3)
Yes	224	131 (58.5)	60 (26.8)	33 (14.7)	40 (17.8)	120 (53.6)	64 (28.6)	158 (70.5)	38 (17.0)	28 (12.5)
**Drinking**
Never	619	338 (54.6)	169 (27.3)	112 (18.1)	160 (25.8)	360 (58.2)	99 (16.0)	454 (73.3)	92 (14.9)	73 (11.8)
Yes	173	98 (56.6)	51 (29.5)	24 (13.9)	33 (19.1)	89 (51.4)	51 (29.5)	111 (64.2)	43 (24.8)	19 (11.0)

PA, physical activity.

### Intergroup comparison of PASE scores and cognitive reaction time in older adults

Table 2 shows the differences between the PASE scores and reaction time of older adults in different age groups. There was a significant difference in the scores of leisure PA between the younger and the older elderly (*p* < 0.01), and there was no significant difference in the scores of housework and work-related PA. In addition, there was no significant difference in cognitive reaction time between the younger and the older elderly (60–74 years: 1.07 ± 0.30 s, ≥75 years: 1.08 ± 0.32 s).

**Table 2:** Comparison of PASE score and reaction time in participants of different ages.

	**60~74 years (*n =* 692)**	**≥75 years (*n =* 100)**	**Z**	**T**	***P*-value**
Leisure PA Score	8.57 (0.00,171.00)	25.71 (0.00,91.57)	5.63		0
Housework PA Score	45.00 (0.00,141.00)	45.00 (0.00,101.00)	−0.27		0.79
Work-related PA Score	0.00 (0.00,120.00)	0.00 (0.00,30.00)	−1.91		0.06
Reaction Time (s)	1.07 ± 0.30	1.08 ± 0.32		−0.59	0.95

PA, physical activity.

The results of the comparison of PASE scores and reaction time of older adults by gender are shown in Table 3. There was a significant difference in the scores of housework between men and women (*p* < 0.01), while the scores of leisure PA and work-related PA did not have a significant difference. Additionally, there was no statistically significant difference in cognitive reaction time between male and female older adults (male: 1.06 ± 0.29 s and female: 1.09 ± 0.32 s).

**Table 3:** Comparison of PASE score and reaction time in participants of different genders.

	**Male (*n =* 384)**	**Female (*n =* 408)**	**Z**	**T**	***P*-value**
Leisure PA Score	8.57 (0.00,171.00)	8.57 (0.00,171.00)	−0.19		0.85
Housework PA Score	50.00 (0.00,141.00)	35.00 (0.00,121.00)	−2.84		0
Work-related PA Score	0.00 (0.00,21.00)	0.00 (0.00,120.00)	−0.11		0.91
Reaction Time (s)	1.06 ± 0.29	1.09 ± 0.32		−1.27	0.2

PA, physical activity.

### The difference in cognitive reaction time in older adults with different types of PA

The cognitive reaction times of elderly subjects with different levels of leisure PA, housework, and work-related PA are shown in Table 4. Using multi-level one-way ANOVA analysis, we could find that there was a significant difference in the reaction time of the elderly with different leisure PA levels, and there was also a significant difference in the reaction time of the elderly with different work-related PA levels. Additionally, after Turkey's *post hoc* analysis, we could distinguish the specific significant differences between these groups.

**Table 4:** The reaction time of participants with different levels of physical activity.

		**Reaction Time (s)**	***P*-value**	***F***
Leisure PA Level	Low	1.11 ± 0.32	0.00	8.1
	Moderate	1.05 ± 0.30*		
	High	0.99 ± 0.28*		
Housework PA Level	Low	1.09 ± 0.31	0.11	0.65
	Moderate	1.07 ± 0.31		
	High	1.05 ± 0.28		
Work-related PA Level	Low	1.09 ± 0.30	0.00	6.48
	Moderate	1.03 ± 0.32^#^		
	High	0.99 ± 0.28^#^		

PA, physical activity; *Compared with “Low” Leisure PA Level, significant difference on reaction time (p < 0.01); ^#^Compared with “Low” Work-related PA Level, significant difference on reaction time (*P* < 0.01).

For leisure PA, the results showed that the cognitive reaction time of older adults in the “Low” level leisure PA group (1.11 ± 0.32 s) was significantly longer than that in the “Moderate” (1.05 ± 0.30 s, *p* < 0.01) and the “High” level leisure PA groups (0.99 ± 0.28 s, *p* < 0.01). There was no significant difference between the “Moderate” and the “High” level leisure PA groups.

For housework PA, although the average cognitive reaction time of the elderly with a “Moderate” level (1.07 ± 0.31 s) and “High” level (1.05 ± 0.28 s) was less than that of the “Low” level (1.09 ± 0.31 s), there was no significant difference among the three groups after using the *post hoc* analysis.

For work-related PA, the results showed that the cognitive reaction time of old adults in the “Low” work-related group (1.09 ± 0.30 s) was significantly longer than that in the “High” (0.99 ± 0.28 s) and the “Moderate” work-related PA groups (1.03 ± 0.32 s, *p* < 0.01). There was no significant difference between the “Moderate” and the “High” level work-related PA groups.

## Discussion

The study categorized PA and explored the effect of the level of each type of PA on cognitive reaction time in older adults providing an important reference for PA to delay cognitive decline in older adults. Not only leisure PA but also active participation in work-related PA can shorten the reaction time of older adults. Moreover, there is no significant difference in the effect of moderate and high levels of PA on the improvement of cognitive reaction time in older adults. To avoid the negative effects of higher levels of PA, older adults are encouraged to maintain a moderate level of leisure PA and work-related PA daily as much as possible according to their abilities.

The descriptive statistics of the demographic and PA of the subjects in this study suggested that older adults are not physically active enough in their leisure time, and more than 50% of them are at a low level of leisure PA. This indicates that most older adults are less physically active and still spend most of their time in a sedentary and less active state, which is also consistent with other studies. Only half of older Australians are physically active enough (25), and only 20% of older Americans are even more physically active (26), while in South Korea, a neighboring country to China, 80% of 60- to 70-year-olds and 90% of those aged 70 years and older do not engage in moderate to high PA (27). This study showed that 55% of Chinese older adults do not engage in moderate to high levels of leisure PA, a lower percentage than in developed countries, but this percentage is likely to increase further as the economy develops, so we need to pay more attention to the phenomenon of insufficient PA among older adults. At the same time, work-related PA accounts for a relatively small proportion of PA among older adults, with more than 70% of older adults having no or little involvement in work-related PA, which is in line with the current reality in China. Older adults have retired after the age of 60 years, and few continue to work, more often taking on household tasks, such as childcare, cleaning, grocery shopping, and cooking. Additionally, this study shows that most older adults have a high level of housework, with only 24% doing little housework in general.

The level of PA in older adults decreases with age (28). However, the present study showed that the level of leisure PA increased in the older elderly compared to the younger elderly, and the older elderly did not reduce leisure PA due to declining physical function. This may be related to the higher education level of the older elderly subjects in this study, who generally attach more importance to physical health and actively participate in various types of physical activities on a daily basis. The reaction times of older adults in this study also did not increase with age, which is different from previous studies that considered age as a risk factor for cognitive function (29), presumably also due to the higher level of education in the older age group, which is a protective factor for cognitive function (30), offsetting the negative effect of age. In addition, there was no significant difference in the cognitive reaction time between men and women in this study, indicating that gender did not affect cognitive function in older adults. However, the level of housework was higher in men than in women, probably because the PASE used in this study only answered “yes/no” participation in housework, while items with higher weight, such as carrying things, cleaning the yard, and repairing, were often performed by men.

The positive effect of leisure PA on cognitive function in older adults was also verified in this study. Spirduso conducted pioneering research on the relationship between PA and cognitive function in older adults. The difference in the cognitive processing speed between the subjects was determined by comparing the reaction time and it was found that older adults with exercise routines had faster reaction times than the average older adults (24). Several subsequent studies have also found that older adults who are physically active have better cognitive performance than those who are sedentary and less active (10, 20, 21). Older adults in this study who usually maintained moderate-to-high levels of leisure PA also had significantly faster reaction speeds than those with low levels of leisure PA. Frequent participation in leisure PA can exercise balance and physical function, reshape the brain, and strengthen synaptic connections in neural networks, thereby delaying the decline of cognitive function in older adults.

This study did not find any improvement in cognitive function in older adults from housework, but a study has found that gardening can improve memory, logical thinking, and communication skills in older adults (31). This study did not separately analyze the effect of flower gardening and watering on the reaction time of older adults. Moreover, in the Chinese revised version of the PASE used in the study, some of the entries are still not suitable for Chinese older adults, such as cleaning the yard (most older adults live in buildings and do not have a yard), which may cause a decrease in the validity of the assessment of housework.

In the PASE, the work-related PA of older adults was mainly participation in jobs that required sitting, standing, walking, or running. The main work-related PA of older adults was participation in street and community volunteer activities, such as patrols. This study found that moderate to high levels of work-related PA can be good for improving cognitive function in older adults. Work-related physical activities involve not only physical activities but also cognitive and social activities. Cognitive (32, 33) and social activities (22) can also benefit cognitive function in older adults. Some studies have also shown that participation in volunteer activities contributes to the development of cognitive reserve in older adults and can slow down the rate of cognitive aging (22). Participation of older adults in volunteer work, in which walking or standing PA improves cardiorespiratory fitness, improves cerebrovascular function, and prevents neuropathological changes (33). In addition, the need of the elderly to communicate with others contributes to the maintenance of their verbal and memory skills.

These findings suggest that older adults should be encouraged to participate in voluntary activities organized by the street or community after retirement, which can reflect their self-worth and slow down cognitive decline. If the elderly are usually burdened with household chores, they can choose to temporarily put aside their chores, such as child care, grocery shopping, and cooking on weekends, to participate in some volunteer activities of interest and get out of the house to communicate more with the outside world. Instead of letting simple and repetitive chores take up most of the time and energy of the elderly, they should make full use of their leisure time for physical exercise, reduce the time spent on watching television (TV), reading newspapers, and other sedentary activities, and do active physical activities in the morning or after dinner every day.

This is a cross-sectional study, and it is difficult to clarify the causal relationship between PA and cognitive reaction time, which has certain limitations. In addition, measuring PA by questionnaires is not precise enough, although it is suitable for large-scale population surveys. In addition, cognitive reaction time is a common index to assess cognitive function, but its assessment of cognitive function is not comprehensive enough.

In future studies, we hope to conduct longitudinal surveys to clarify the effects of PA on cognitive reaction time in older adults through dynamic and long-term observations. The recommended amount of PA to delay cognitive decline in older adults can also be given through more accurate PA measurements. In addition, cognitive function in older adults is a multidimensional indicator, and the effects of PA on cognitive function in older adults should be explored in terms of attention, memory, and responsiveness.

## Conclusion

Leisure PA and work-related PA have positive implications for improving cognitive reaction time in older adults. Cognitive reaction times were significantly shorter in older adults who maintained moderate and high levels of leisure PA and work-related PA. The effect of high levels of leisure PA and work-related PA on improving cognitive reaction time in older adults was not significantly different from that of moderate levels. To avoid other negative effects of high levels of PA, moderate levels of leisure PA and work-related PA are recommended for older adults to slow down the decline of cognitive function.

## Data availability statement

The original contributions presented in the study are included in the article/Supplementary material, further inquiries can be directed to the corresponding author.

## Ethics statement

The studies involving human participants were reviewed and approved by Medical Ethics Committee of Tsinghua University. The patients/participants provided their written informed consent to participate in this study.

## Author contributions

JL: conceptualization, resources, and supervision. YL and XH: methodology and writing—original draft preparation. YL: formal analysis and visualization. ZT and HZ: investigation. YL and JL: writing—review and editing. All authors have read and agreed to the published version of the manuscript.

## Conflict of interest

The authors declare that the research was conducted in the absence of any commercial or financial relationships that could be construed as a potential conflict of interest.

## Publisher's note

All claims expressed in this article are solely those of the authors and do not necessarily represent those of their affiliated organizations, or those of the publisher, the editors and the reviewers. Any product that may be evaluated in this article, or claim that may be made by its manufacturer, is not guaranteed or endorsed by the publisher.

## References

[1] World Health Organization. World Health Organization launches new initiative to address health needs of a rapidly ageing population. Indian J Med Sci. (2004) 58:411–2. Available online at: https://www.ncbi.nlm.nih.gov/pubmed/15902777 15902777

[2] Akimov AV Gemueva KA Semenova NK. The seventh population census in the PRC: results and prospects of the country's demographic development. Her Russ Acad Sci. (2021) 91:724–35. 10.1134/S1019331621060083 35125844 PMC8807378

[3] Kim EJ Yoon YS Hong S Son HY Na TY Lee MH. Retinoic acid receptor-related orphan receptor alpha-induced activation of adenosine monophosphate-activated protein kinase results in attenuation of hepatic steatosis. Hepatology. (2012) 55:1379–88. 10.1002/hep.25529 22183856

[4] Tian LP Deshmukh A Prasad N Jang YY. alcohol increases liver progenitor populations and induces disease phenotypes in human IPSC-derived mature stage hepatic cells. Int J Biol Sci. (2016) 12:1052–62. 10.7150/ijbs.15811 27570479 PMC4997049

[5] Brigola AG Ottaviani AC Alexandre TD Luchesi BM Pavarini SCI. Cumulative effects of cognitive impairment and frailty on functional decline, falls and hospitalization: a four-year follow-up study with older adults. Arch Gerontol Geriat. (2020) 87:104005. 10.1016/j.archger.2019.104005 31901850

[6] Li CL Chang HY Stanaway FF. Combined effects of frailty status and cognitive impairment on health-related quality of life among community dwelling older adults. Arch Gerontol Geriat. (2020) 87:1039999. 10.1016/j.archger.2019.103999 31874329

[7] Lee Y Kim J Chon D Lee KE Kim JH Myeong S. The effects of frailty and cognitive impairment on 3-year mortality in older adults. Maturitas. (2018) 107:50–5. 10.1016/j.maturitas.2017.10.006 29169580

[8] World Health O. China Country Assessment Report on Ageing and Health. Geneva: World Health Organization (2015).

[9] Kirk-Sanchez NJ McGough EL. Physical exercise and cognitive performance in the elderly: current perspectives. Clin Interv Aging. (2014) 9:51. 10.2147/CIA.S39506 24379659 PMC3872007

[10] Rogers RL Meyer JS Mortel KF. After reaching retirement age physical-activity sustains cerebral perfusion and cognition. J Am Geriatr Soc. (1990) 38:123–8. 10.1111/j.1532-5415.1990.tb03472.x 2299115

[11] Angevaren M Aufdemkampe G Verhaar HJJ Aleman A Vanhees L. Physical activity and enhanced fitness to improve cognitive function in older people without known cognitive impairment. Cochrane Db Syst Rev. (2008) 2:3. 10.1002/14651858.CD005381.pub3 18646126

[12] Cordova C Silva VC Moraes CF Simoes HG Nobrega OT. Acute exercise performed close to the anaerobic threshold improves cognitive performance in elderly females. Braz J Med Biol Res. (2009) 42:458–64. 10.1590/S0100-879X2009000500010 19377796

[13] Ruscheweyh R Willemer C Kruger K Duning T Warnecke T Sommer J. Physical activity and memory functions: an interventional study. Neurobiol Aging. (2011) 32:1304–19. 10.1016/j.neurobiolaging.2009.08.001 19716631

[14] Erickson KI Voss MW Prakash RS Basak C Szabo A Chaddock L. Exercise training increases size of hippocampus and improves memory. P Natl Acad Sci USA. (2011) 108:3017–22. 10.1073/pnas.1015950108 21282661 PMC3041121

[15] Scherder E Scherder R Verburgh L Konigs M Blom M Kramer AF. Executive functions of sedentary elderly may benefit from walking: a systematic review and meta-analysis. Am J Geriat Psychiat. (2014) 22:782–91. 10.1016/j.jagp.2012.12.026 23636004

[16] DeLoss DJ Watanabe T Andersen GJ. Improving vision among older adults: behavioral training to improve sight. Psychol Sci. (2015) 26:456–66. 10.1177/0956797614567510 25749697 PMC4441618

[17] Washburn RA Smith KW Jette AM Janney CA. The physical-activity scale for the elderly (pase) - development and evaluation. J Clin Epidemiol. (1993) 46:153–62. 10.1016/0895-4356(93)90053-4 8437031

[18] Carvalho A Rea IM Parimon T Cusack BJ. Physical activity and cognitive function in individuals over 60 years of age: a systematic review. Clin Interv Aging. (2014) 9:661–82. 10.2147/CIA.S55520 24748784 PMC3990369

[19] Buchman AS Boyle PA Yu L Shah RC Wilson RS Bennett DA. Total daily physical activity and the risk of AD and cognitive decline in older adults. Neurology. (2012) 78:1323–9. 10.1212/WNL.0b013e3182535d35 22517108 PMC3335448

[20] Dustman RE Shearer DE Emmerson RY. Eeg and event-related potentials in normal aging. Prog Neurobiol. (1993) 41:369–401. 10.1016/0301-0082(93)90005-D 8210412

[21] Muscari A Giannoni C Pierpaoli L Berzigotti A Maietta P Foschi E. Chronic endurance exercise training prevents aging-related cognitive decline in healthy older adults: a randomized controlled trial. Int J Geriatr Psych. (2010) 25:1055–64. 10.1002/gps.2462 20033904

[22] Adam S Bonsang E Grotz C Perelman S. Occupational activity and cognitive reserve: implications in terms of prevention of cognitive aging and Alzheimer's disease. Clin Interv Aging. (2013) 8:377–90. 10.2147/CIA.S39921 23671387 PMC3650883

[23] Yu HJ Zhu WM Qiu J Zhang CG. Physical activity scale for elderly (PASE): a cross-validation study for Chinese older adults. Med Sci Sport Exer. (2012) 44:647.

[24] Spirduso WW. Reaction and movement time as a function of age and physical-activity level. J Gerontol. (1975) 30:435–40. 10.1093/geronj/30.4.435 1141674

[25] Lim K Taylor L. Factors associated with physical activity among older people - a population-based study. Prev Med. (2005) 40:33–40. 10.1016/j.ypmed.2004.04.046 15530578

[26] Topolski TD LoGerfo J Patrick DL Williams B Walwick J Patrick MB. The rapid assessment of physical activity (RAPA) among older adults. Prev Chron Dis. (2006) 3:A118. Available online at: https://www.ncbi.nlm.nih.gov/pmc/articles/PMC1779282 16978493 PMC1779282

[27] Akarolo-Anthony SN Adebamowo CA. Prevalence and correlates of leisure-time physical activity among Nigerians. BMC Public Health. (2014) 14:529. 10.1186/1471-2458-14-529 24885080 PMC4050994

[28] Washburn RA McAuley E Katula J Mihalko SL Boileau RA. The physical activity scale for the elderly (PASE): evidence for validity. J Clin Epidemiol. (1999) 52:643–51. 10.1016/S0895-4356(99)00049-9 10391658

[29] Gaucher J Kinouchi K Ceglia N Montellier E Peleg S Greco CM. Distinct metabolic adaptation of liver circadian pathways to acute and chronic patterns of alcohol intake. P Natl Acad Sci USA. (2019) 116:25250–9. 10.1073/pnas.1911189116 31757851 PMC6911183

[30] Le Carret N Lafont S Letenneur L Dartigues JF Mayo W Fabrigoule C. The effect of education on cognitive performances and its implication for the constitution of the cognitive reserve. Dev Neuropsychol. (2003) 23:317–37. 10.1207/S15326942DN2303_1 12740188

[31] McPhee JS French DP Jackson D Nazroo J Pendleton N Degens H. Physical activity in older age: perspectives for healthy ageing and frailty. Biogerontology. (2016) 17:567–80. 10.1007/s10522-016-9641-0 26936444 PMC4889622

[32] Krueger KR Wilson RS Kamenetsky JM Barnes LL Bienias JL Bennett DA. Social engagement and cognitive function in old age. Exp Aging Res. (2009) 35:45–60. 10.1080/03610730802545028 19173101 PMC2758920

[33] Fratiglioni L Paillard-Borg S Winblad B. An active and socially integrated lifestyle in late life might protect against dementia. Lancet Neurol. (2004) 3:343–53. 10.1016/S1474-4422(04)00767-7 15157849

